# Effect of Inorganic N Top Dressing and *Trichoderma harzianum* Seed-Inoculation on Crop Yield and the Shaping of Root Microbial Communities of Wheat Plants Cultivated Under High Basal N Fertilization

**DOI:** 10.3389/fpls.2020.575861

**Published:** 2020-10-23

**Authors:** María Illescas, M. Belén Rubio, Víctor Hernández-Ruiz, María E. Morán-Diez, A. Emilio Martínez de Alba, Carlos Nicolás, Enrique Monte, Rosa Hermosa

**Affiliations:** ^1^Spanish-Portuguese Institute for Agricultural Research (CIALE), Department of Microbiology and Genetics, University of Salamanca, Salamanca, Spain; ^2^Spanish-Portuguese Institute for Agricultural Research (CIALE), Department of Botany and Plant Physiology, University of Salamanca, Salamanca, Spain

**Keywords:** bacterial composition, fungal composition, chemical fertilization, bulk soil, rhizosphere, root endosphere

## Abstract

Wheat crop production needs nitrogen (N) for ensuring yield and quality. High doses of inorganic N fertilizer are applied to soil before sowing (basal dressing), with additional doses supplied along the cultivation (top dressing). Here, a long-term wheat field trial (12 plots), including four conditions (control, N top dressing, *Trichoderma harzianum* T34 seed-inoculation, and top dressing plus T34) in triplicate, was performed to assess, under high basal N fertilization, the influence of these treatments on crop yield and root microbial community shaping. Crop yield was not affected by top dressing and *T. harzianum* T34, but top dressing significantly increased grain protein and gluten contents. Twenty-seven-week old wheat plants were collected at 12 days after top dressing application and sampled as bulk soil, rhizosphere and root endosphere compartments in order to analyze their bacterial and fungal assemblies by 16S rDNA and ITS2 high-throughput sequencing, respectively. Significant differences for bacterial and fungal richness and diversity were detected among the three compartments with a microbial decline from bulk soil to root endosphere. The most abundant wheat root phyla were Proteobacteria and Actinobacteria for bacteria, and Ascomycota and Basidiomycota for fungi. An enrichment of genera commonly associated with soils subjected to chemical N fertilization was observed: *Kaistobacter*, *Mortierella*, and *Solicoccozyma* in bulk soil, *Olpidium* in rhizosphere, and *Janthinobacterium* and *Pedobacter* in root endosphere. Taxa whose abundance significantly differed among conditions within each compartment were identified. Results show that: (i) single or strain T34-combined application of N top dressing affected to a greater extent the bulk soil bacterial levels than the use of T34 alone; (ii) when N top dressing and T34 were applied in combination, the N fertilizer played a more decisive role in the bacterial microbiome than T34; (iii) many genera of plant beneficial bacteria, negatively affected by N top dressing, were increased by the application of T34 alone; (iv) bulk soil and rhizosphere fungal microbiomes were affected by any of the three treatments assayed; and (v) all treatments reduced *Claroideoglomus* in bulk soil but the single application of T34 raised the rhizosphere levels of this mycorrhizal fungus.

## Introduction

Wheat is one of the most important crops worldwide, with figures like a harvested area of 214.3 million ha and a global production of 734 million tons in 2018 (FAOSTAT, 2020). Given the fact that wheat grain provides about one-fifth of both calories and proteins to human diet, there is, therefore, a need for increasing the production of this crop in order to feed the world’s-growing population (International Wheat Genome Sequencing Consortium [IWGSC], 2018). Conventional extensive agriculture has an absolute requirement of nitrogen (N) for ensuring the yield and high quality of wheat crops (Zörb et al., 2018). However, it is well known that this and other widespread cereal crops use only 30–40% of the applied N fertilizers, while the rest remains unused causing severe environmental pollution (Rockström et al., 2009; Curci et al., 2017). Although the EU is suggesting a reduction in N fertilization to quantities of 170 kg/ha/year, in countries like Spain, where 2.4 million ha are devoted to this crop, this figure can still reach as high as 500 kg/ha/year. It is a common practice in Spain to apply 240 kg/ha as basal nitrogen fertilizer and to add a higher quantity as top dressings along the wheat crop.

Several studies have reported a wide range of beneficial effects of the microbiome members on plants, including disease suppression, priming of the plant immune system leading to the induction of systemic resistance, increased nutrient acquisition, increased tolerance to abiotic stresses or adaptation to environmental variations (Hassani et al., 2018). It is now evident that the root system provides many more traits than just anchorage and uptake of nutrients and water, and therefore all the interconnected factors that influence the complex ecosystem of the rhizosphere, considering it as an integrated whole, including numerous and multiple kinds of microorganisms that interact in various ways need to be taken into account (Mendes et al., 2011). Besides the well-known mycorrhizal fungi, N-fixing bacteria, and growth-promoting bacteria, plant microbiomes include a high diversity of microorganisms that become apparent when comparing microbial species and strains even at the level of the genotypes from a same species (Vandenkoornhuyse et al., 2002; Bulgarelli et al., 2012; Peiffer et al., 2013; Rossmann et al., 2020).

Advances in next-generation sequencing (NGS) technologies have marked the beginning of a new era in gathering information on the genetic repertoires of microbial communities (Fricker et al., 2019). The Proteobacteria, mostly alpha and beta classes, usually dominate in root-associated samples. Other major bacterial groups that are often present in the roots include Actinobacteria, Acidobacteria, Cyanobacteria, Firmicutes, FCB (Fibrobacteres-Chlorobi-Bacteroidetes), particularly Bacteroidetes and Gemmatimonadetes, and PVC (Planctomycetes-Verrucomicrobia-Chlamydiae), especially Planctomycetes and Verrucomicrobia (Philippot et al., 2013; Turner et al., 2013). A large number of research reports have explored the fungal communities associated with plant roots, revealing a staggering diversity of fungi, mainly belonging to the two major phyla Ascomycetes and Basidiomycetes (Porras-Alfaro and Bayman, 2011; Rossmann et al., 2020; Wang et al., 2020). Fungal communities are not randomly assembled but instead appear to be specifically filtered by their plant host which recruits a particular microbial consortium to adapt to the environmental conditions at a microscale (Lê Van et al., 2017). At least three distinct microbiomes thriving at the root-soil interface have been identified (Bulgarelli et al., 2012; Hirsch and Mauchline, 2012), depending on whether they belong to bulk soil, rhizosphere or endosphere. In almost all cases, an apparent decrease in the diversity of species was recorded from the rhizosphere to the endosphere, indicating that exists a strong habitat filtering mechanism and that it may shape the composition of each microbiome compartment (Vandenkoornhuyse et al., 2015). It has been described that the bacterial rhizosphere changes much more than the bulk soil community in wheat cropping systems (Donn et al., 2015). Moreover, soil nutrient availability constitutes a driving factor in shaping the wheat endophytic bacterial microbiome (Robinson et al., 2015), although the use of N fertilization negatively affects bacterial assemblages in the wheat rhizosphere (Kavamura et al., 2018).

Most *Trichoderma* spp. have been linked to biocontrol against plant pathogenic fungi, oomycetes, and even nematodes (Medeiros et al., 2017; Debbi et al., 2018). Moreover, rhizosphere competent strains have proved to be beneficial for plants (Hermosa et al., 2012). *Trichoderma* species are frequently found as common inhabitants of the soil and the rhizosphere, and even though many of them may become facultative endophytes, the number of truly endophytic *Trichoderma* spp. is scarce (Bae et al., 2009; Carrero-Carrón et al., 2018). In addition to rhizosphere colonization, nutrient uptake facilitation and plant growth promotion (Hermosa et al., 2012; Samolski et al., 2012), the application of *Trichoderma* strains may also affect the soil bacterial and fungal communities in a pH- and N supply dependent manner, respectively (Zhang et al., 2018). It has been reported that biofertilizers based on *Trichoderma* strains when used alone or in combination with organic fertilizers (compost) provoke changes in the rhizosphere microbial community of crop plants (Zhang et al., 2013; Pang et al., 2017; Ros et al., 2017; Qiao et al., 2019). Specifically, *Trichoderma* spp. have been directly related to the increased levels of Acidobacteria detected in different agricultural soils such as those from maize and black pepper (Saravanakumar et al., 2017; Umadevi et al., 2017; Singh et al., 2018). The combined application of *Trichoderma*, and other beneficial microorganisms such as *Bacillus*, to crop soils fosters the recruitment of other plant beneficial bacteria and fungi in the rhizosphere (Wang et al., 2019).

The previous studies showed positive effects of *T. harzianum* T34 on tomato plant growth under greenhouse conditions (Rubio et al., 2017) and also the ability of this strain to increase wheat systemic defense after culturing under *in vitro* conditions (Rubio et al., 2019). However, little is known about the effects caused by the application of *Trichoderma* or N-based fertilizers on the microbiota of wheat plants under field conditions, and whether the microbial communities are randomly assembled or specifically filtered by the host plant to create a particular microbial assemblage to meet the new requirements of the environment. The conventional agronomic practices for wheat crop in the Spanish region of Castile and Leon include the application of high doses of N fertilizer to the soil before the sowing (basal) and along the cultivation (top dressing). We lack of a complete understanding of how bacterial and fungal communities are structured in crop plants, how fertilization practices can alter microbial communities, how such practices might affect microbe performance, and how they are in turn linked to their potential microbial preys. Here, the aim of our work has been to assess the diversity and structure of both bacterial and fungal communities in the root system of wheat crop plants subjected to three different treatments (top dressing, *T. harzianum* T34, and strain T34 plus top dressing) in order to explore, under high basal N dosage (control), the influence of inorganic N top dressing and *Trichoderma* application in the microbiome distribution at the bulk soil, rhizosphere and endosphere compartments.

## Materials and Methods

### Field Wheat Experiment and Sample Collection

A field trial was performed in Ventosa de la Cuesta (Valladolid, Spain), a region with continental Mediterranean climate and an average annual temperature and precipitation of 12.5°C and 415 mm, respectively. The experimental field was preceded by fallow for 1 year and this last by a barley crop. The trial was carried out over 1 year from 2018 to 2019 and included 12 experimental plots containing four conditions (C1, C2, C3, and C4) with three replicates in a randomized complete block design (Supplementary Figure S1). Each experimental plot had 12.75 m^2^ (8.5 × 1.5 m) with a plantation framework of 425 seeds/m^2^, corresponding approximately to 240 kg seeds/ha, and using wheat of the Berdun R variety.

The four conditions were designed as follows: C1 (control: soil amended with basal chemical fertilizer), C2 (soil amended with basal chemical fertilizer and two top dressing applications), C3 (soil amended with basal chemical fertilizer and strain *T. harzianum* T34), and C4 (soil amended with basal chemical fertilizer, and both strain T34 and two top dressing applications). Following conventional agronomic practices in this region, 2 days before sowing 240 kg/ha of NPK 8-15-15 and 30 kg/ha of KCl were applied as basal chemical fertilization. The first top dressing application was performed 12 weeks after sowing with the 60% of N requirement (157 kg/ha of calcium nitrate, CAN) in conditions C2 and C4, and the second CAN dosage was supplied similarly 27 weeks after sowing with the 40% of total N requirement (105 kg/ha) in these two conditions. *Trichoderma harzianum* CECT 2413 (Spanish Type Culture Collection, Valencia, Spain), also referred to as strain T34, was grown on potato dextrose agar medium (PDA, Difco Laboratories, Detroit, MI, United States) and spores were harvested as previously described (Rubio et al., 2017). Strain T34 was seed-coating applied at a concentration of 2 × 10^6^ conidia/seed in the C3 and C4 conditions. The procedure was carried on through the addition of 15 mL of a T34 suspension (6.7 × 10^8^ conidia/mL) and 10 mL of a commercial Arabic gum solution (Pelikan, Barcelona, Spain) to plastic bags containing 250 g of wheat seeds and subsequent manual mixing. The seed-inoculated bags were kept open for 20 h in a laminar flow cabinet for drying. Furthermore, 15 mL of sterile water and 10 mL of Arabic gum solution were added to each bag with seeds for C1 and C2 conditions.

Sampling process was carried out at 27 weeks after sowing (12 days after applying the second top dressing where indicated). Soil and wheat samples were collected from five spots selected within each plot (assayed condition) and were considered as a single sample. Three biological replicates per condition were considered. The five sample spots were uniformly selected across plots (Supplementary Figure S1). From each spot a total of 20 plants were harvested by digging a hole (up to about 30 cm deep and 15 cm wide) around the pool of plants with a trench shovel in order to collect the whole root system of the plants (bulk soil and rhizosphere) as well as the areal part. The 20 plants from each of the five spots were carefully placed in one plastic bag and labeled with the condition, replicate, and sampling plot. In addition, soil samples were collected from each hole for chemical analysis. Approximately, 100 g of fine earth was collected from each hole with a hand shovel, once the pool of plants was taking out, and placed in a 50 mL sterile tube. The soil samples from the five spots per plot were combined in a single sample and three replicates per condition were considered. Soil and plant samples were taken to the laboratory for processing. The crop was harvested on June 28th, 2019 and the grains were used to calculate crop yield (kg/ha), macro- and microelements content, and quality parameters such as protein and gluten contents.

### Root Sample Preparation

In order to carry out the microbiota analysis, the whole root system of the plants (set of 100 plants per plot) was processed to isolate three different samples: (i) bulk soil, considered as the soil at a distance of 2–6 cm of the root surface; (ii) rhizosphere, considered as the loosely adhering soil from the root system; and (iii) root endosphere, considered as the inside of surface-disinfected roots. Samples were prepared according to the methodology previously described (D’Amico et al., 2018; Yamamoto et al., 2018) with slight modifications. Briefly, for each plant set, 10 g of bulk soil was uniformly hand-collected, taking care not to disturb any root, and placed in a 50 mL sterile tube, frozen in liquid N, and stored at −80°C. Once the soil attached to the roots was removed, the root systems of the set of plants were laid on a flat bench as a unit and transversally cut with scissors. From these roots, 3.5 g were collected, cut into segments and washed twice with 20 mL PBS-S buffer (130 mM NaCl, 7 mM Na_2_HPO_4_, 3mMNaH_2_PO_4_, pH 7.0, 0.02% Silwet L-77), by shaking at 180 rpm in a 50 mL tube for 20 min. Washed roots were transferred to a 50 mL sterile tube and the remained liquid was filtered throught nylon. The obtained liquid, about 38 mL, was centrifuged at 3200 rpm and 4°C for 15 min. The generated pellet, called as the rhizosphere, was frozen in liquid N, and stored at −80°C. The roots were subsequently washed by shaking as described above once in 35 mL of 2% commercial sodium hypochlorite and three times in 35 mL of PBS-S buffer. Then, the roots were transferred to a 50 mL sterile tube with 35 mL PBS-S buffer, sonicated for 20 min with a water bath sonicator at 40 kHz (Model 5510, Branson Ultrasonics Corporation, Danbury, CT, United States), and washed again in 35 mL of PBS buffer using the same procedure described above. Roots were dried on 50 mm diameter Whatman filter paper, transferred to a 50 mL sterile tube, and then frozen in liquid nitrogen for storage at −80°C. The three sample types obtained were used for DNA extraction.

### Chemical Properties of Soil and Grains

All measurements were quantified by the IRNASA’s analytical service (CSIC, Salamanca, Spain), apart from protein and gluten contents in wheat grain. For the soil, the 12 sample sets of 0.5 kg were sieved and an aliquot of 100 g used for determination of pH and content in CaCO_3_, carbon (C), organic matter, N and phosphorus (P). For the 12 sets of pooled wheat grain, the samples were powdered and 500 mg used for quantification of C, N, macro- and microelements.

The pH of the soil was determined in a soil/water suspension (1:2.5, w/v ratio) with a glass electrode. N and organic C contents, expressed as percentage (g per 100 g sampled material), were determined by dry combustion (Dumas, 1831) in a CN628 automatic carbon-nitrogen analyzer (LECO Instruments S.L., Madrid, Spain) following the manufacturer’s instructions. Organic C data were used to calculate the organic matter percentage. The content of inorganic carbon was determined as CaCO_3_ with a Bernard calcimeter. The available P in soil samples was estimated by extraction with sodium bicarbonate (Olsen et al., 1954).

The content of macro- [sulphur (S), P, magnesium (Mg), potassium (K) and calcium (Ca)] and microelements [iron (Fe), manganese (Mn), zinc (Zn) and copper (Cu)] was determined by mineralization in a mixture of nitric acid and hydrogen peroxide (4:1 v/v) using an Ethos Up High Performance Microwave Digestion System (Milestone, Sorisole, Italy). Samples were subjected to the microwave heating with a temperature ramp ranging from room temperature to 200°C for 40 min, followed by maintaining at 200°C for 15 min. After cooling, solutions were quantitatively transferred into 25 mL volumetric flasks and brought up to volume with ultrapure water. The content of such elements was analyzed by ICP-OES (Inductively Coupled Plasma Optical Emission Spectrometry, iCAP 6300 DUO, Thermo Electron Corporation, Rugby, United Kingdom), as previously described (Jiménez et al., 2019).

Protein and wet gluten contents were determined in 0.5 kg of pooled wheat grains per plot by near-infrared spectroscopy (NIR) technology (Chen et al., 2017), using a portable Zeltex ZX50 NIR analyzer (Zeltex Inc., Hagerstown, MD, United States). Values were expressed as percentage.

### DNA Extraction, PCR Amplifications and Illumina Sequencing

DNA was extracted from all the 36 sample sets, 12 from each soil, rhizosphere and root endosphere compartments. Root endosphere samples were previously lyophilized and ground to a fine powder with a sterilized mortar and a pestle. Total DNA of bulk soil and rhizosphere samples was extracted using the FastDNA Spin Kit for Soil (MP Biomedical LLC, Irvine, CA, United States) and that of root endosphere samples using the NucleoSpin Plant Kit (Macherey-Nagel, Düren, Germany), following manufacturer’s instructions. Each sample had three replicates in our experiment, and the triplicate DNA samples were pooled. Approximately 30 ng of DNA for each sample was sent to the Genomics Unit (Parque Científico de Madrid, Madrid, Spain) for amplification, library preparation and sequencing.

The 16S rRNA and ITS region were used to determine bacterial and fungal communities, respectively, in all the 36 sample sets from soil, rhizosphere and root endosphere. DNA concentration was determined in the samples using Quant-IT PicoGreen reagent (ThermoFisher Scientific, Waltham, MA, United States). Purified DNAs (3 ng) and the universal primers 341f (5′-CCTACGGGNGGCWGCAG-3′) and 785r (5′-GACTACHVGGGTATCTAATCC-3′) were employed to amplify the V3-V4 region of the bacterial 16S rRNA gene, and the primers ITS86F (5′-GTGAATCATCGAATCTTTGAA-3′) and ITS4 reverse (5′-TCCTCCGCTTATTGATATGC-3′) were used to amplify the ITS2 region of the fungal ITS. The PCR mix was prepared as previously described (Zhang et al., 2018). The PCR thermal cycling program consisted of initial denaturation at 98°C for 30 s, followed by 20 (for 16S) or 21 (for ITS) cycles of denaturation at 95°C for 10 s, annealing at 55°C for 20 s and extension at 72°C for 20 s, and a final extension step at 72°C for 2 min. Each sample was amplified in triplicate and subsequently the PCR products were pooled. PCR products (approximately 450 and 300 pb in size for bacterial and fungal samples, respectively) included extension tails which allowed sample barcoding and the addition of specific Illumina sequences in a second low cycle number PCR. The obtained amplicons were validated and quantified by a Bioanalyzer, and an equimolecular pool of 16S and ITS PCR products was purified using AMPure beads and titrated by quantitative PCR using the “Kapa-SYBR FAST qPCR kit for Light Cycler 480” and a reference standard for quantification. The pool of amplicons was denatured before seeding on a flowcell of an Illumina Miseq platform at a density of 10 pM, and the cluster were formed and sequenced using a “MiSeq Reagent Nano Kit v2” and a 2 × 250 pair-end sequencing run. Illumina sequencing resulted in a total of 3,621,101 reads for 16S and 4,019,719 reads for ITS that passed Illumina quality control (Supplementary Tables S1, S2). The obtained bacterial 16S and fungal ITS sequences data are available at the Sequence Read Archive (SRA), operated by the National Center for Biotechnology Information (NCBI), under the accession number PRJNA639567.

### Bioinformatics Processing and Taxonomy Assignment

Sequence quality was evaluated for raw forward and reverse Illumina ITS and 16S reads with FastQC (Andrews, 2010). Preprocessing and quality control filtering, operational taxonomic unit (OTU) clustering, taxonomy assignment and construction of the abundance tables were performed using USEARCH v11.1 (Edgar, 2010). Sequences which could not be assembled, singletons, chimeras, and sequences with a low quality score were discarded.

For both bacterial and fungal communities, OTUs were clustered with at least 97% similarity threshold using UPARSE-OTU algorithm (Edgar, 2013) and were taxonomically assigned using the GreenGenes v13.5 (DeSantis et al., 2006) and UNITE USEARCH/UTAX release for fungi version 18.11.2018 (Kõljalg et al., 2013), a database specifically modified for USEARCH pipeline, respectively. Only taxonomic annotations with a 97% confidence estimate as provided by the SINTAX algorithm (Edgar, 2016) command were accepted. Taxonomy assignment provided the available annotation of each OTU to the different taxonomy levels (kingdom, phylum, class, order, family, genus, and species). The low abundance OTUs were eliminated from the OTU table if they did not have a total of at least 10 counts across all the dataset, moreover, OTUs assigned to mitochondria (o__Rickettsiales/f__mitochondria) and chloroplasts (p__Cyanobacteria/c__Chloroplast) were removed before downstream analysis. Taxonomic prediction was explored. A phylogenetic tree was generated using *cluster_tree* command from USEARCH v11.1.

### Statistical Analyses

#### Metagenomics Data

All metagenomic data analyses were conducted in RStudio v3.6.2 (R Core Team, 2019). Rarefaction curves were constructed for each sample individually per compartment (bulk soil, rhizosphere and root endosphere) and condition type (C1–C4) using *rarefy_even_depth* command from *phyloseq* package (McMurdie and Holmes, 2013). Redundancy analysis (RDA) was performed based on *vegan* package (Oksanen et al., 2015) to evaluate the taxonomic structure of bacterial and fungal communities and to correlate them with compartment and condition type using Hellinger distance. A hierarchical clustering was performed to examine whether there were clusters between samples and relate them to the environmental conditions using the euclidean distance and the complete linkage method.

Sample richness and evenness were estimated using total number of observed OTUs and the alpha-diversity indices [Chao1 and abundance-based coverage estimator (ACE), Shannon, Simpson, Pielou and Phylogenetic Diversity (PD)]. The PD index was calculated using the *picante* package (Kembel et al., 2010), the rest of indices were calculated using the *phyloseq* package. Kruskal–Wallis sum-rank test was used to compare difference in medians of alpha-diversity indices across the three compartments and the four conditions types. Wilcoxon rank-sum test was further employed to test for pairwise significant differences. Bacterial and fungal beta-diversity was estimated according to the Bray–Curtis and un/weighted UniFrac distances from the abundance matrix across samples. A Permutational Multivariate Analysis of Variance (PERMANOVA) test was performed to determine whether bacterial and fungal communities were significantly influenced by compartment and condition types, with 999 permutations, and a multivariate pairwise test for pairwise comparisons using the *adonis* command from *vegan* package and *pairwise.adonis* from *PairwiseAdonis* (Martinez-Arbizu, 2017), respectively. Principal coordinates analysis (PCoA) based on these beta-diversity distances were used to visualize the dissimilarities among the compartments and condition types.

The relative abundance of taxa at the phylum, family, genus and species levels was calculated and depicted by stacked barplots. The differential abundance testing was conducted using ALDEx2 (Fernandes et al., 2013) in order to explore whether the abundance for bacteria and fungi data varied at the genus level among compartments and among conditions within a given compartment. ALDEx2 uses the centered log-ratio (clr) transform which ensures that the data is scale invariant and compositional consistent. Before carrying out this analysis, a filtering was performed, excluding those OTUs with a relative abundance of less than 0.001%. While ALDEx2 provides both parametric and non-parametric statistical tests, only non-parametric test results are reported in this study, Kruskal–Wallis test followed by Wilcoxon test were used for pairwise comparisons. Significance was measured based on the Benjamini–Hochberg corrected *P-*value for both tests (significance threshold, *P* < 0.05). In pairwise comparisons, ALDEx2 also provides an effect size and a 95% confidence interval (95% CI).

Finally, the linear discriminant analysis (LDA) effect size (LEfSe) method from Huttenhower Lab (Segata et al., 2011), which is based on the Kruskal–Wallis sum-rank test for comparison classes, was also used to identify genera significantly different among compartments and, within compartments, among conditions. An LDA threshold score > 4.0 for compartments and >2.0 for conditions, and a significance *P* < 0.05 threshold for conditions as well as a sample normalization to 1 M, which is usually applied for metagenomic data in which the relative abundances are taken into account, were used. Different LEfSe-generated taxonomic cladograms from phylum to genus were produced.

#### Agronomic Data

All data were collected from three biological replicates. The homogeneity of variances and normality tests were performed by Levene’s and Shapiro–Wilk’s tests. The data of soil parameter, agronomic traits, and micro- and macroelements content agreed with the parametric statistics assumptions were further analyzed. One-way and two-way ANOVA were performed followed by a post-hoc Tukey’s test using the IBM SPSS Statistics for Windows, version 25 (IBM Corp., Armonk, NY, United States) and setting confidence intervals of 95%.

## Results

To explore the soil parameters existing at the time of collecting the microbiome samples, soil samples were also collected, pH measured, and content in organic matter, CaCO_3_, C, N, and P was analyzed. Non-variability among conditions was detected (Table 1). In addition, there was not significant effect of combining T34 and top dressing upon the outcome of these values.

**TABLE 1:** Soil parameters’ analysis in samples from a field wheat trial under four different conditions collected at 27 weeks after sowing and 12 days after second top dressing application where corresponded.

Parameters*^a^*	Conditions*^b^*						
	C1	C2	C3	C4	*P*_*T34*_*^c^*	*P*_*TD*_	*P*_*T34* × *TD*_
pH	7.81 ± 0.45^a^	7.71 ± 0.83^a^	8.04 ± 0.43^a^	7.07 ± 0.50^a^	ns	ns	ns
CaCO_3_ (%)	1.05 ± 0.74^a^	1.06 ± 0.92^a^	1.48 ± 0.96^a^	0.27 ± 0.47^a^	ns	ns	ns
C (%)	0.54 ± 0.11^a^	0.56 ± 0.14^a^	0.53 ± 0.17^a^	0.59 ± 0.04^a^	ns	ns	ns
Organic matter (%)	0.93 ± 0.19^a^	0.97 ± 0.25^a^	0.91 ± 0.30^a^	1.02 ± 0.06^a^	ns	ns	ns
N (%)	0.07 ± 0.01^a^	0.06 ± 0.01^a^	0.07 ± 0.01^a^	0.07 ± 0.00^a^	ns	ns	ns
C/N ratio	8.27 ± 1.82^a^	9.00 ± 1.71^a^	7.93 ± 3.15^a^	8.17 ± 0.35^a^	ns	ns	ns
P (ppm)	21.72 ± 8.55^a^	19.06 ± 4.01^a^	25.09 ± 2.65^a^	19.07 ± 6.11^a^	ns	ns	ns

*^a^For each parameter, values are means of three plots by treatment (n = 3). Values in the same row with same letter are not significantly different according to Tukey’s test (P > 0.05). ^b^The four conditions, under high basal chemical N fertilization, were as follow: C1, control; C2, two applications of calcium nitrate as top dressing (TD); C3, T. harzianum T34 (T34); and C4, T34 plus two applications of calcium nitrate as TD. ^c^Significant effects were determined by a two-way analysis of variance (ANOVA) for T34, TD, and the interactions between both factors (T34 × TD) (Tukey’s test, P < 0.05). ns, no statistical differences.*

In order to determinate associations between microbiome data and crop traits, parameters such as final crop production, specific weight, and protein and gluten contents were calculated in the grain samples that were harvested from this wheat trial (Table 2). No statistical differences were observed for yield values among the four conditions (*P* > 0.05), although compared to control (C1) conditions C2, C3, and C4 tended to increase final production. Gluten values recorded for conditions C2 and C4, both supplemented with CAN top dressing, were significantly higher than those of C1. Regarding grain protein content, significant higher percentages were only observed for CAN top dressing (C2) when compared to C1. A two-way ANOVA statistical analysis of the data from the four crop parameters indicated above showed that the top dressing application increased protein (*P* = 0.024) and gluten (*P* = 0.017) in grain when top dressing-applied and not applied conditions were compared. These parameters were not significantly modified by the T34 application relative to the T34-unapplied conditions, and non-significant changes for these four parameters were detected in the double T34 and top dressing interaction. In terms of micro- and macroelements content in harvested grain (Table 3), no significant differences among the tested conditions were detected for N, Ca and the four microelements analyzed. Compared to control (C1), the single application of T34 (C3) significantly increased the Mg and K contents but reduced that of C, while the top dressing application alone (C2) or in combination with T34 (C4) increased the S content. Interestingly, the combined application of T34 and top dressing (C4) decreased the content of P when compared to the single application of T34 (C3). Despite the effect caused by the T34 and top dressing applications on the content of some elements, only the Mg content was significantly affected by the combination of both factors (*P* < 0.05).

**TABLE 2:** Agronomic traits of wheat grain harvested from a field trial under four different conditions.

Traits*^a^*	Conditions*^b^*						
	C1	C2	C3	C4	*P*_*T34*_*^c^*	*P*_*TD*_	*P*_*T34* × TD_
Yield (kg/ha)	6154.0 ± 188.09^a^	6613.0 ± 362.93^a^	7098.5 ± 931.26^a^	6950.3 ± 722.39^a^	ns	ns	ns
Wet gluten (%)	24.30 ± 0.28^b^	27.00 ± 0.82^a^	25.45 ± 0.78^ab^	26.57 ± 1.18^a^	ns	*	ns
Proteins (%)	10.67 ± 0.05^b^	11.28 ± 0.15^a^	10.90 ± 0.16^ab^	11.07 ± 0.29^ab^	ns	*	ns
Specific weight (kg/hl)	80.90 ± 0.57^a^	80.93 ± 0.31^a^	80.80 ± 0.00^a^	81.03 ± 0.32^a^	ns	ns	ns

*^a^For each parameter, values are means of three plots by condition (n = 3). Values in the same row with same letter are not significantly different according to Duncan’s test (P > 0.05). ^b^The four conditions, under high basal chemical N fertilization, were as follows: C1, control; C2, two applications of calcium nitrate as top dressing (TD); C3, T. harzianum T34 seed-inoculation (T34); and C4, T34 plus two applications of calcium nitrate as TD. ^c^Significant effects were determined by a two-way analysis of variance (ANOVA) for T34, TD, and the interactions between both factors (T34 × TD) (Duncan’s test, P < 0.05). ns, no significant differences.*

**TABLE 3:** Measurement of micro- and macroelements content on wheat grain harvested from the field trial at 27 weeks after sowing and 12 days after second top dressing application where corresponded.

Elements*^a^*	Conditions*^b^*							
		C1	C2	C3	C4	*P*_*T34*_*^c^*	*P*_*TD*_	*P*_*T34* × *TD*_
Macro-elements	C	45.53 ± 0.14^b^	45.59 ± 0.08^b^	45.14 ± 0.12^a^	45.31 ± 0.22^ab^	*	ns	ns
	N	1.85 ± 0.03^a^	1.96 ± 0.02^a^	1.88 ± 0.08^a^	1.96 ± 0.07^a^	ns	*	ns
	Ca	0.44 ± 0.02^a^	0.44 ± 0.02^a^	0.43 ± 0.01^a^	0.44 ± 0.02^a^	ns	ns	ns
	K	3.22 ± 0.11^a^	3.20 ± 0.10^a^	3.51 ± 0.10^b^	3.33 ± 0.15^ab^	*	ns	ns
	Mg	1.20 ± 0.02^a^	1.17 ± 0.01^a^	1.25 ± 0.03^b^	1.16 ± 0.02^a^	ns	*	*
	P	2.51 ± 0.10^ab^	2.49 ± 0.05^ab^	2.75 ± 0.11^b^	2.48 ± 0.13^a^	ns	*	ns
	S	1.47 ± 0.05^a^	1.64 ± 0.01^b^	1.55 ± 0.08^ab^	1.62 ± 0.04^b^	ns	*	ns
Micro-elements	Cu	3.22 ± 0.30^a^	2.91 ± 0.25^a^	3.15 ± 0.13^a^	2.82 ± 0.25^a^	ns	*	ns
	Fe	42.85 ± 21.91^a^	61.79 ± 29–40^a^	45.80 ± 13.72^a^	50.25 ± 17.16^a^	ns	ns	ns
	Mn	22.63 ± 1.12^a^	22.32 ± 2–26^a^	23.43 ± 1.68^a^	22.18 ± 1.82^a^	ns	ns	ns
	Zn	11.30 ± 1.65^a^	11.89 ± 1.11^a^	12.53 ± 1.25^a^	12.32 ± 0.68^a^	ns	ns	ns

*^a^Macroelements: C and N = %; Ca, K, Mg, P, and S = g/kg of grain. Microelements = mg/kg of grain. Data are the mean of three plots for each condition (n = 3). Values in the same row with different letter are significantly different according to Tukey’s test (P > 0.05). ^b^The four conditions, under high basal chemical N fertilization, was as follow: C1, control; C2, two applications of calcium nitrate as top dressing (TD); C3, T. harzianum T34 (T34); and C4, T34 plus two applications of calcium nitrate as TD. ^c^Significant effects were determined by a two-way analysis of variance (ANOVA) for T34, TD, and the interactions between both factors (T34 x TD) (Tukey’s test, P < 0.05), ns: no statistical differences.*

### Bacterial Microbiome Assembly in Wheat Crop Plants Under Different Conditions

#### Exploratory Analysis of Bacterial Libraries

We obtained 3,621,101 raw reads for V3–V4 region from the Illumina Miseq of the 36 samples (99.6% of them with Phred score of 20). After filtering the 3,130,161 clean sequences, a total of 2,541,261 high-quality reads were obtained with an average of 70,608 ± 24,328 per sample (Supplementary Table S1). The sequence reads were clustered into 5,984 OTUs at 97% identity and, after removing low abundance OTUs, a total of 4,990 OTUs were used to analyze bacterial diversity and composition.

The RDA used to explore the differences across the 36 samples showed that RDA1 (compartment) and RDA2 (condition) explained 48.7% (*P* < 0.001) and 9.6% (*P* < 0.001) variability, respectively. Moreover, the composition of communities was significantly affected by the factor compartment (*P* < 0.001) but not by the factor tested condition (*P* = 0.267). In any case, further bacterial composition analyses allowed to study the effect of the condition factor within each compartment. Considering the degree of bacterial taxonomy prediction, 97% of the OTUs were assigned at phylum level and the percentage was gradually reduced at class (91.9), order (76.8) and family (44.2) level, until it reached 13.6 and 0.24% at genus and species level, respectively.

#### Diversity of Bacterial Communities

**FIGURE 1:**
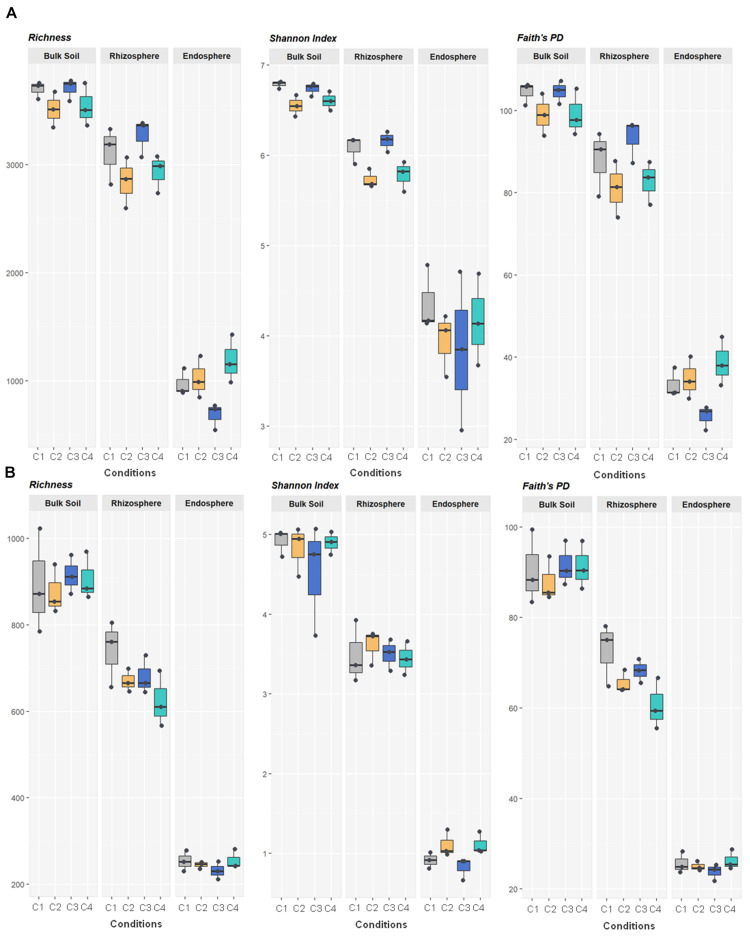
Bacterial **(A)** and fungal **(B)** variety in 36 samples of wheat crop plants under four different conditions, using the total number of OTUs observed (richness) and the indices of Shannon and Faith’s Phylogenetic Diversity. The four conditions, under high basal inorganic N fertilization, were as follows: C1, control; C2, two applications of calcium nitrate as top dressing (TD); C3, *T. harzianum* T34 seed-inoculation (T34); and C4, T34 plus two applications of calcium nitrate as TD. Whiskers represent the minimum and maximum values. All other points are contained within the box, and the bar represents the median. For all metrics, bulk soil, rhizosphere and root endosphere samples were significantly separated (Kruskal–Wallis test, *P* < 0.001).

**FIGURE 2:**
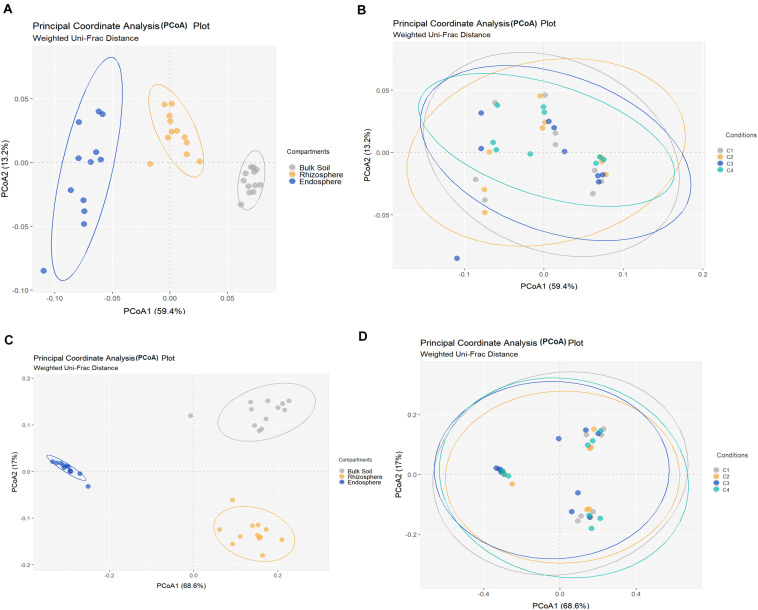
Principal coordinates analysis (PCoA) of bacterial **(A,B)** and fungal **(C,D)** community structures in different samples of wheat crop plants, based on the weighted UniFrac distance model. Bacterial **(A)** and fungal **(C)** PCoAs show significant segregation among bulk soil, rhizosphere and root endosphere samples. Bacterial **(B)** and fungal **(D)** PCoAs show no separation among conditions [C1, control; C2, two applications of calcium nitrate as top dressing (TD); C3, *T. harzianum* T34 seed-inoculation (T34); and C4, T34 plus two applications of calcium nitrate as TD] samples. Permutational multivariate PERMANOVA based on distance matrices (Adonis), *P* = 0.001.

All metrics used to calculate richness and/or biodiversity, including observed OTUs, Shannon index and Faith’s PD (Figure 1A), exhibited similar trends among the three compartments. Values of Shannon index for bulk soil and rhizosphere samples were >5.5 which was indicative of a moderate-high bacterial diversity, while values of Simpson index were close to 1 for these two compartment samples which indicated dominance of some taxons. Moreover, root endosphere samples pointed out to a moderate diversity (Shannon index: 3 to under 5). A significantly decrease in bacterial alpha-diversity, estimated by Shannon and Faith’s PD index, from bulk soil to root endosphere was observed (Kruskal–Wallis test, *P* < 0.001), and there were not differences among conditions (*P* > 0.05). Regarding beta-diversity PCoA, similar PCoA plots were observed using weighted/unweighted UniFrac and Bray-Curtis distance models. All of them revealed significant separation among compartments (*P* < 0.001) but no segregation among tested conditions (*P* > 0.9). The index of weighted UniFrac distance (Figures 2A,B) was able to explain the 72.6% variability reached, such distance being only significantly affected by the factor compartment (PERMANOVA, *P* < 0.001; and for all pairwise comparisons post-hoc *Adonis P* < 0.001, *P* adjusted 0.003). Moreover, root endosphere samples displayed the highest dispersion degree.

#### Composition of Bacterial Communities

**FIGURE 3:**
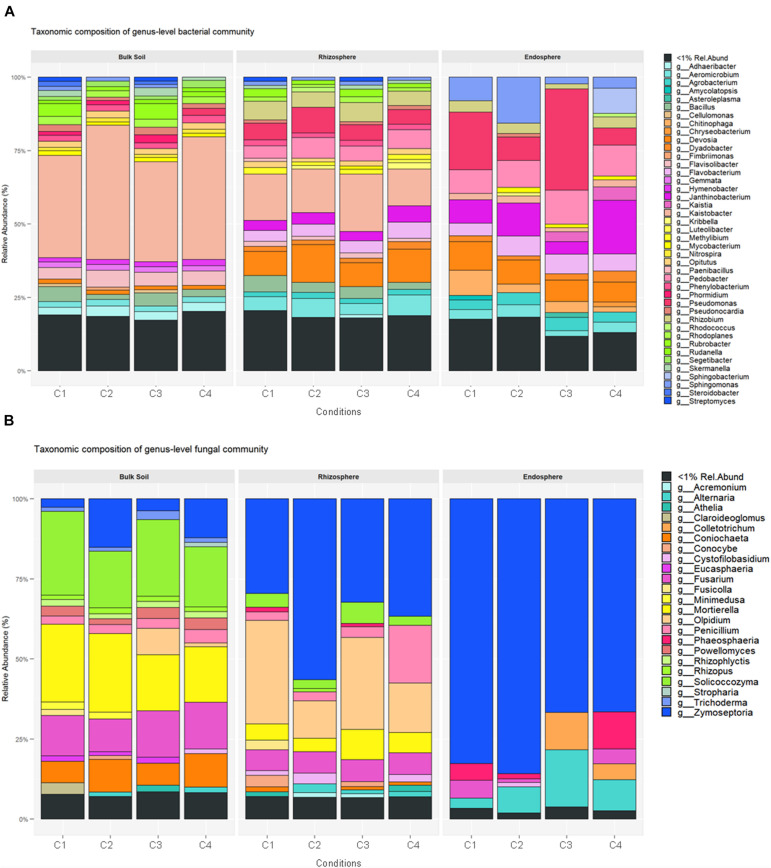
Average of relative abundance of bacteria **(A)** and fungi **(B)** in bulk soil, rhizosphere and root endosphere samples of wheat crop plants under four different conditions [C1, control; C2, two applications of calcium nitrate as top dressing (TD); C3, *T. harzianum* T34 seed-inoculation (T34); and C4, T34 plus two applications of calcium nitrate as TD]. Relative abundance at genus level was used for comparisons, and mean value of the three sample replicates for each condition is shown.

Considering the relative abundance of taxonomically assigned OTUs, members of the phyla Proteobacteria (74.3–16.5%) and Actinobacteria (63.1–12.7%) dominated in the 36 samples, followed by Bacteroidetes (18.3–4.8%), Acidobacteria (14.4–0.2%), Gemmatimonadetes (6.9–0.1%) and Chloroflexi (5.2–0.2%). The wide range of these percentages indicates that there is a high variability among the 36 samples. At phylum and family levels, bulk soil and rhizosphere samples showed closer composition patterns than those from root endosphere (Supplementary Table S3). Nevertheless, the bacterial composition differed in relative abundance among the three compartments analyzed. Actinobacteria and Proteobacteria were the most relatively abundant bacteria in root endosphere, while Bacteroidetes increased their presence in bulk soil. The relative abundance of Acidobacteria, Gemmatimonadetes, Chloroflexi, Verrucomicrobia, Planctomycetes and Cyanobacteria decreased from bulk soil to root endosphere. A summary of the genera with assigned names and relative abundance higher than 1% is shown in Figure 3A.

**FIGURE 4:**
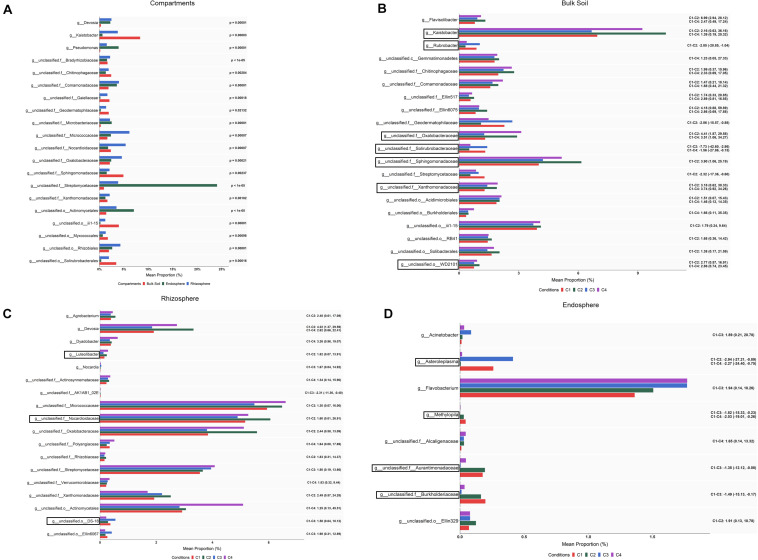
Bacterial taxa showing differential abundance among compartments **(A)** and conditions within bulk soil **(B)**, rhizosphere **(C)** and root endosphere **(D)** of wheat crop plants. Abundance is shown as mean proportion and was calculated by ALDEx2 at the genus level. Significance for compartments is based on corrected Kruskal–Wallis test (*P* < 0.05) and for conditions in effect size (95% CI) (*n* = 3). Conditions were: C1, control; C2, two applications of calcium nitrate as top dressing (TD); C3, *T. harzianum* T34 seed-inoculation (T34); and C4, T34 plus two applications of calcium nitrate as TD. For panels **(A,B)**, only the 20 most abundant taxa are shown. Black boxes refer to the taxa identified by both ALDEx2 and LEfSe analyses. For panels **(B–D)**, significant pairwise comparisons with median effect size values and in brackets their lower and upper limits are indicated. Black boxes refer to the taxa identified by both ALDEx2 and LEfSe analyses.

When abundance differences among compartments were explored at the genus level by ALDEx2 analysis, 231 taxa showing differential abundance (corrected Kruskal–Wallis, *P* < 0.05) were identified: 195 corresponded to endospore vs. bulk soil; 163 to rhizosphere vs. bulk soil; and 108 to endosphere vs. rhizosphere (Supplementary Table S4). Based on the effect size (95% CI), still a larger number of taxa whose abundance differed among compartments were observed. Bearing in mind these pairwise comparison results, it can be pointed out that the levels of genera such as *Pedobacter*, *Janthinobacterium*, *Agrobacterium*, *Flavobacter* or *Chitinophaga* were gradually increased from bulk soil to root endosphere, while *Kaistobacter* followed an opposite direction showing the highest levels in the bulk soil. Results also showed that genera such as *Devosia*, *Rhizobium*, and *Sphingomonas* were increased in rhizosphere and root endosphere. The 20 bacterial taxa with the highest values of average relative abundance (mean proportions) are presented in Figure 4A.

Differential abundance among compartments was also analyzed at the genus level by the LEfSe method, and 41 bacterial taxa showing differences in abundance (LDA > 4, *P* < 0.05) were identified (Supplementary Table S5). In order to better understand the changes occurring from phylum to genus, a LEfSe taxonomic cladodram was generated (Supplementary Figure S2A). Different considerations could be taken into account for the factor compartment: (i) most changes due to conditions occurred in bulk soil; (ii) the phyla Acidobacteria, Gemmatimonadetes, Chloroflexi, Verrucomicrobia, and Plactomycetes in bulk soil, and Enterobactereriaceae in endosphere, were increased, and (iii) the order Rhizobiales increased in the rhizosphere. At the genus level, 11 taxa showed to be differentially more abundant (LDA > 4, *P* < 0.05) in one of the compartments (Supplementary Figure S2B). They were distributed in this way: (i) *Kaistobacter* and one member from each of the following taxa: family Sphingomonadaceae, order Solirubrobacterales and the Acidobacteria order iii1-15, in bulk soil; (ii) one member of order Rhizobiales and another from family Nocardioidaceae, in rhizosphere; and (iii) *Janthinobacterium* and the FCB *Pedobacter*, and three members belonging to the order Actinomycetales and the families Streptomycetaceae and Enterobacteriaceae, in root endosphere.

Although our ALDEx2 and LEfSe analyses performed at the genus level could not identify taxa differing in abundance among conditions through all three compartments, many taxa were identified when such differences were explored within each of the three compartments by ALDEx2 (Supplementary Table S4). After pairwise comparisons, between conditions C2, C3, or C4 and C1 (control), differences in abundance (effect size, 95% CI) were detected in 81, 16 and 9 taxa for bulk soil, rhizosphere and root endosphere samples, respectively. As expected, most of these taxa corresponded to genera annotated as unclassified for the three compartments. Results show that most changes associated to conditions occurred in bulk soil. Taxa showing significant differences in abundance among conditions within bulk soil, rhizosphere and root endosphere are respectively included in Figures 4B–D. As they were many in bulk soil, only the 20 taxa with the highest values of average relative abundance (mean proportions) were depicted. According to the abundance differences detected in bulk soil: (i) application of the top dressing alone (C2) caused a decrease in the levels of *Streptomyces*, *Cellulomonas*, *Nonomuraea*, *Rubrobacter*, *Haliangium* and *Brevibacillus*, but increased those of *Aeromicrobium*, *Kaistobacter*, *Gemmatimonas*, *Luteolibacter*, *Flavisolibacter*, and *Opitutus*; (ii) no changes were associated to the single application of strain T34 (C3); and (iii) the combined application of top dressing and strain T34 reduced the levels of *Williamsia*, *Haliangium* and *Steroidobacter*, but increased those of *Kaistobacter*, *Gemmatimonas*, *Luteolibacter*, *Flavisolibacter*, *Janthinobacterium*, and *Lysobacter*. The differences in abundance shared by conditions C2 and C4, for at least five genera, indicate that they are due to top dressing. The rhizospheric levels of *Devosia*, *Luteolibacter*, and *Agrobacterium* in the condition C2, *Nocardia* in C3, and *Dyadobacter* in C4 were significantly increased when compared with those of C1. The fact that increased levels of *Devosia* were also detected in C4 and that a taxa of the order Actynomicetales was only differentially increased in that condition, is an example of the particular effects caused by the combined application of top dressing and strain T34 on the different rhizospheric bacterial taxa (Figure 4C). The lowest number of taxa showing changes in abundance associated with conditions was recorded in the root endosphere (Figure 4D). Compared to the control condition, the endosphere changes showed that the single application of strain T34 (C3) increases the levels of *Acinetobacter* and when applied in combination with top dressing (C4) reduces *Methylopila* and *Asteroleplasma*, although lower levels of the latter genus were also observed when top dressing is applied alone (C2) (Figure 4D).

**FIGURE 5:**
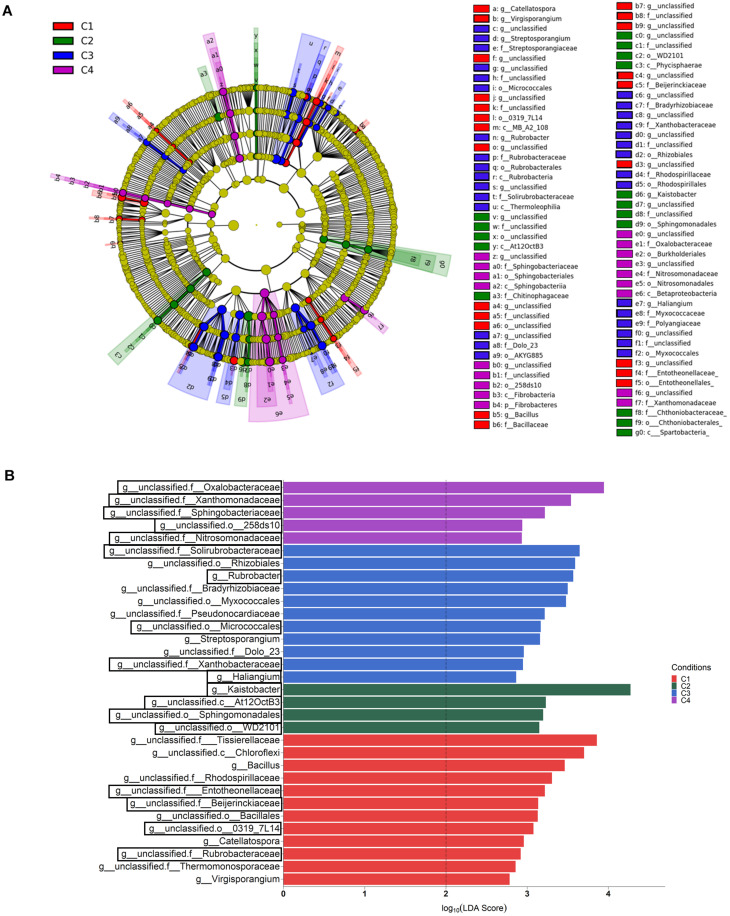
Bulk soil bacterial taxa overrepresented in the four tested conditions on wheat crop plants after LEfSe analysis at the genus level. Taxonomic cladogram showing differences from phylum to genus level **(A)**, and bacterial taxa with LDA > 2 (*P* < 0.05) **(B)** (*n* = 3). Conditions were: C1, control; C2, two applications of calcium nitrate as top dressing (TD); C3, *T. harzianum* T34 seed-inoculation (T34); and C4, T34 plus two applications of calcium nitrate as TD. In the cladogram, dot size is proportional to taxon abundance and letters refer to the taxa listed on the right. In the barplot, black boxes refer to the taxa identified by both LEfSe and ALDEx2 analyses.

**FIGURE 6:**
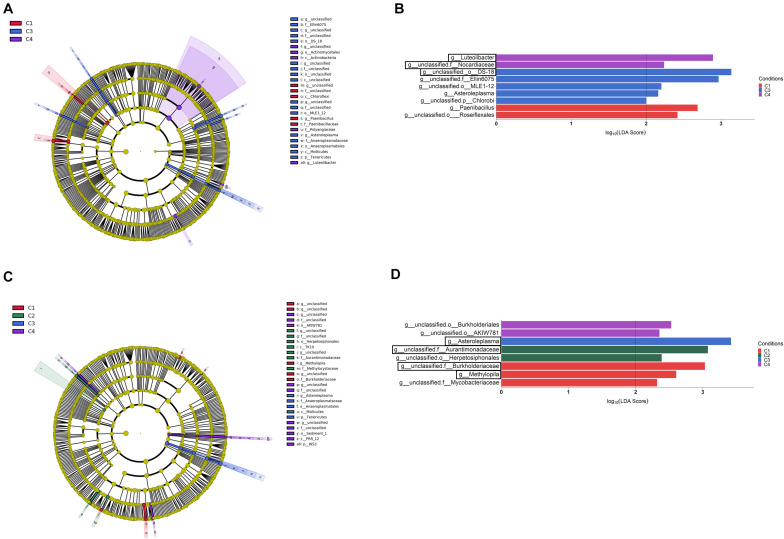
Rhizosphere **(A,B)** and root endosphere **(C,D)** bacterial taxa overrepresented in the four tested conditions on wheat crop plants after LEfSe analysis at the genus level. Taxonomic cladograms showing differences from phylum to genus level in the rhizosphere **(A)** and the root endosphere **(C)**, and bacterial taxa with LDA > 2 (*P* < 0.05) in the rhizosphere **(B)** and the root endosphere **(D)** (*n* = 3). Conditions were: C1, control; C2, two applications of calcium nitrate as top dressing (TD); C3, *T. harzianum* T34 seed-inoculation (T34); and C4, T34 plus two applications of calcium nitrate as TD. In cladograms, dot size is proportional to taxon abundance and letters refer to the taxa listed on the right. In barplots, black boxes refer to the taxa identified by both LEfSe and ALDEx2 analyses.

When the category condition was explored by LEfSe for each compartment (LDA > 2, *P* < 0.05), a total of 87, 27 and 21 taxa showing differential abundance in bulk soil, rhizosphere and endosphere, respectively, were identified (Figures 5, 6). Changes occurring from phylum to genus are shown in cladograms (Figures 5A, 6A,C), and taxa identified at the genus level according to their differential abundance among conditions are separately displayed (Figures 5B, 6B,D). For bulk soil, in addition to several unclassified genus taxa (Figure 5B), increased levels of the genera *Bacillus*, *Catellatospora* and *Virgisporangium* in condition C1, *Kaistobacter* in C2, and *Rubrobacter*, *Streptosporangium*, and *Haliangium* in C3, were detected. The analysis of rhizosphere taxa identified that the abundance of 12 of them was affected by the factor condition (Figure 6B), with increases for *Paenibacillus* in C1, *Asteroleplasma* in C3 and *Luteolibacter* in C4. In the endosphere, *Methylopila* in C1 and *Asteroleplasma* in C3 were two of the eight taxa with increased levels detected under any of the conditions (Figure 6D).

### Fungal Microbiome Assembly in Wheat Crop Plants Under Different Conditions

#### Exploratory Analysis of Fungal Libraries

We obtained 4,019,719 raw reads for the ITS2 region from the Illumina Miseq of the 36 samples (98.5% of them with Phred score of 20). After filtering the 3,397,598 clean sequences, a total of 3,386,201 high-quality reads were obtained with an average of 94,061 ± 15,261 per sample (Supplementary Table S2). The sequence reads were clustered into 3,497 OTUs at 97% identity and, after removing low abundance OTUs, a total of 2,056 OTUs were used to analyze fungal diversity and composition.

The RDA used to explore the differences across the 36 fungal libraries showed that compartment and condition variables explained 48.7% (*P* < 0.001) and 9.6% (*P* < 0.001) variability, respectively. As observed for bacterial samples, the separation of fungal ones was affected by the factor compartment (*P* < 0.001) but not by the factor condition (*P* = 0.11). Subsequent fungal composition analyses allowed to study the effect of the condition factor within each compartment. It can be pointed out that the predicted degree of the reached fungal taxonomy was low. At phylum level, only 26.4% of the OTUs were assigned, and the percentage decreased at class (19.7%), order (17.2%), family (11.4%), genus (6.91%), and species (1.8%) levels.

#### Diversity of Fungal Communities

Fungal richness and alpha-diversity obtained across the 36 samples (Figure 1B) were only significantly affected by the factor compartment (Kruskal–Wallis test, *P* < 0.001). The lowest richness (total observed OTUs, Chao1, ACE) corresponded to root endosphere samples. Regarding estimated alpha-diversity, three groups were separated by Shannon index values of ca. 1 (root endosphere), above 3 (rhizosphere), and close to 5 (bulk soil), being indicative of low, low-moderate and moderate diversity, respectively (Figure 1B). There was taxa dominance for bulk soil and rhizosphere samples, as supported by Simpson index values close to 1, and this did not occur in root endosphere samples. Similar results were obtained for the beta-diversity estimated by weighted/unweighted UniFrac and Bray-Curtis distance models, confirming that the variable compartment was a significant factor for the spatial separation of the 36 samples in the three groups. The most discriminative PCoA plot was observed for the distance model weighted UniFrac (Figures 2C,D) that explained the reached 85.6% variability, of which 68.6% was due to the component compartment (PERMANOVA, *P* < 0.001; and for all pairwise comparisons *post hoc Adonis P* < 0.001, *P* adjusted 0.003). Moreover, root endosphere samples showed the lowest dispersion degree within a given group (root endosphere vs. bulk soil, *P* = 2.993 10^–10^; and root endosphere vs. rhizosphere, *P* = 1.580 10^–7^).

#### Composition of Fungal Communities

The relative abundance calculated at the different taxonomy levels led to a picture of compositional structure extremely uneven for the three compartments concerned. A total of 46 phyla with a relative abundance > 1% showed differences among samples, where the seven most abundant predicted phyla were Ascomycota, Basidiomycota, Olpidiomycota, Chytridiomycota, Mortierellomycota, Glomeromycota and Mucoromycota (Supplementary Table S6). However, their relative abundance differed for the three compartments and ranged as follow: Ascomycota in bulk soil (39.04–20.5%), rhizosphere (35.33–18.1%), and root endosphere (7.48–1.43%); and Basidiomycota in rhizosphere (14.52–4.07%), bulk soil (9.12–3.2%), and root endosphere (0.94–0.02%) samples. Moreover, the phyla Mortierellomycota and Olpidiomycota were increased in bulk soil and rhizosphere samples, respectively. The fungal genera with assigned name and relative abundance higher than 1% are presented in Figure 3B.

**FIGURE 7:**
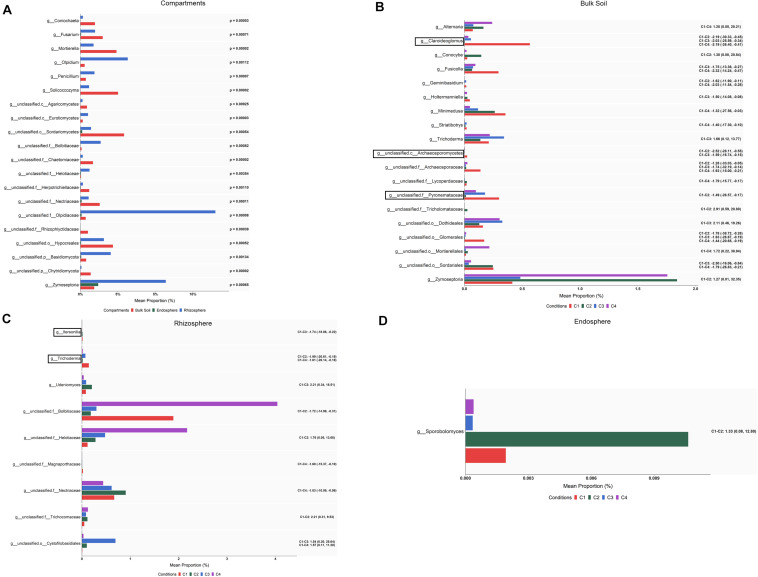
Fungal taxa showing differential abundance among compartments **(A)** and conditions within bulk soil **(B)**, rhizosphere **(C),** and root endosphere **(D)** of wheat crop plants. Abundance is shown as mean proportion and was calculated by ALDEx2 at the genus level. Significance for compartments is based on corrected Kruskal–Wallis test (*P* < 0.05) and for conditions in effect size (95% CI) (*n* = 3). Conditions were: C1, control; C2, two applications of calcium nitrate as top dressing (TD); C3, *T. harzianum* T34 seed-inoculation (T34); and C4, T34 plus two applications of calcium nitrate as TD. For panels **(A,B)**, only the 20 most abundant taxa are shown. For panels **(B–D)**, significant pairwise comparisons with median effect size values and in brackets their lower and upper limits are indicated. Black boxes refer to the taxa identified by both ALDEx2 and LEfSe analyses.

An ALDEx2 analysis of the differences in abundance at the genus level among compartments identified changes (corrected Kruskal–Wallis, *P* < 0.05) in 64 fungal taxa: 51 corresponded to endosphere vs. bulk soil, 31 to endosphere vs. rhizosphere, and 46 to rhizosphere vs. bulk soil (Supplementary Table S7). Lower numbers of taxa whose abundance differed among compartments were detected considering the effect size. Results (effect size, 95% CI) showed that the levels of *Solicoccozyma*, *Mortierella*, *Eucasphaeria*, *Rhizopus*, *Powellomyces*, *Coniochaeta*, *Rhizophlyctis*, and *Trichoderma* decreased from bulk soil to rhizosphere, and many of these were not present in root endosphere. The 20 differential taxa showing the highest percentages of average relative abundance, calculated as mean proportions, are shown in Figure 7A. It can be also observed that the levels of genera such as *Olpidium*, *Penicillium*, and *Zymoseptoria* were increased in rhizosphere, and those of *Fusarium* in bulk soil.

The LEfSe-based differential abundance of fungi was also explored at the genus level among compartments and a total of 41 fungal taxa showing differences (LDA > 4, *P* < 0.001) was identified (Supplementary Table S8 and Supplementary Figure S3). Changes from phylum to genus among compartments are represented in a taxonomic cladogram (Supplementary Figure S3A). The genera *Solicoccozyma* and *Mortierella* as well as one member from each of the following taxa: phylum Ascomycota, order Hypocreales and class Sordariomycetes, were more abundant in bulk soil, while *Olpidium* and a member of phylum Basidiomycota were more abundant in rhizosphere.

When differences in abundance were investigated by ALDEx2 at the genus level within each of the three compartments, several taxa were identified (Supplementary Table S7). After pairwise comparing each condition with the control (C1), 29, 13 and one taxa presented differential abundance (effect size, 95% CI) among some of the compared conditions in bulk soil, rhizosphere and root endosphere, respectively (Figures 7B–D). In the case of bulk soil taxa, only 20 with the highest abundance are shown in Figure 7B. According to the differences detected in bulk soil (effect size, 95% CI) for genera with assigned name (Figure 7B), it can be deduced that: (i) the single application of top dressing (C2) caused a decrease of *Claroideoglomus* and *Geminibasidium* but also an increase of *Conocybe* and *Zymoseptoria* levels; (ii) the single application of strain T34 (C3) reduced the levels of *Claroideoglomus*, *Fusicolla* and *Holtermanniella*, and increased those of *Trichoderma*; and (iii) the combined application of top dressing and strain T34 (C4) also reduced the levels of *Claroideoglomus*, *Fusicolla*, *Geminibasidium*, *Holtermanniella*, *Minimedusa* and *Striatibotrys*, with *Alternaria* being increased. Lower and higher rhizospheric levels of *Trichoderma* and *Udeniomyces* were respectively detected in C2 (Figure 7C). As *Trichoderma* was also decreased in the condition C4, such reduction can be associated with the application of top dressing. In the root endosphere (Figure 7D), only significant changes were observed for the yeast *Sporobolomyces*, which levels were raised by the single application of top dressing.

**FIGURE 8:**
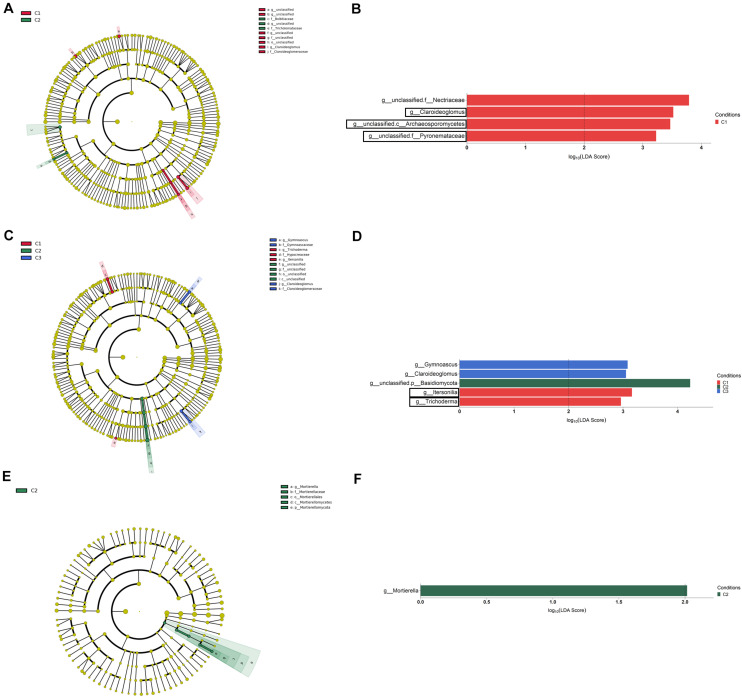
Bulk soil **(A,B)**, rhizosphere **(C,D)**, and root endosphere **(E,F)** fungal taxa overrepresented in the four tested conditions on wheat crop plants after LEfSe analysis at the genus level. Taxonomic cladograms showing differences from phylum to genus level in bulk soil **(A)**, rhizosphere **(C)**, and root endosphere **(E)**, and fungal taxa with LDA > 2 (*P* < 0.05) in bulk soil **(B)**, rhizosphere **(D)**, and the root endosphere **(F)** (*n* = 3). Conditions were: C1, control; C2, two applications of calcium nitrate as top dressing (TD); C3, *T. harzianum* T34 seed-inoculation (T34); and C4, T34 plus two applications of calcium nitrate as TD. In cladograms, dot size is proportional to taxon abundance and letters refer to the taxa listed on the right. In barplots, black boxes refer to the taxa identified by both LEfSe and ALDEx2 analyses.

The LEfSe analysis at the genus level among conditions (LDA > 2, *P* < 0.05) let us to identify a total of four, five and one taxa showing differential abundance in bulk soil, rhizosphere and root endosphere, respectively (Figures 8A–F). Considering the differences observed in genera with assigned name, it can be established that: (i) the levels of *Claroideoglomus* in bulk soil, and *Itersonilia* and *Trichoderma* in rhizosphere were negatively affected by any of the three treatments assayed; (ii) the single application of strain T34 increased the rhizospheric levels of *Gymnoascus* and *Claroideoglomus*; and (iii) the single application of top dressing increased the levels of *Mortierella* in the root endosphere.

## Discussion

A wheat microbiome study was performed in a non-irrigated field trial, under the conventional agronomic practices for this crop in Spain, to explore the influence of inorganic N top dressing, *T. harzianum* T34 and their combination on root microbial community shaping and production traits. Yield results showed that nor did the application of top dressing or strain T34 influence the crop yield. A recent study has reported that low N fertilization increases sweet potato yield, whereas high N fertilization inhibits biological N fixation and produces unintended environmental consequences (Ding et al., 2020). We have seen that the effect of the T34 strain upon the growth of wheat plants is significantly determined by the concentration of chemical N fertilizer (Rubio et al., 2019). Thus, we should not rule out the fact that the basal chemical N dosage (240 kg/ha) could have been so high that led to not improvement in the crop yield for neither top dressing application nor strain T34 seed-inoculation. Even though the absence of a N basal fertilization condition might be considered as a flaw in the experimental design, it is worth noting that the application of high basal N fertilization is a very common practice in wheat intensive production in the region where the trial took place, so the possibility of following the conventional agronomic practices was considered the best and true to customs approach. Soil parameters analyses, including N (%), did not show differences among conditions (Table 1). Although we cannot exclude the possibility that part of the N applied as top dressing could be lost, other part of that N could be uptaken by plants. This statement is based on the positive effects of top dressing on grain gluten and protein contents detected for the conditions C2 and C4 (Table 2). In this sense, a two-way ANOVA showed that the N content of grains, as well as that of Mg, P, S, and Cu, were significantly increased by CAN top dressing application (Table 3), demonstrating in any case the practical value of this treatment. Particularly, the grain gluten content is a highly valuable quality parameter by the flour industry. However, several considerations linked to the use of chemical fertilization on wheat crop should be taken into account as chemical N fertilization costs are high and N is one of the major inputs for intensive production. It is well known that the unused N by plants ends up polluting the environment, and so the adjustment of chemical N fertilizer dosages or even the replacement with biofertilizers are needed goals. Either way, there is still a lack of knowledge upon the use of beneficial organisms such as *Trichoderma* on wheat crops and their effects on fertilization (Meena et al., 2016; Mahato et al., 2018).

Many microbiome studies in wheat cropping systems have been focused on bacterial communities (Donn et al., 2015; Robinson et al., 2015; Rascovan et al., 2016; Kavamura et al., 2018), but the most recent ones include both bacterial and fungal analyses (Friberg et al., 2019; Schlatter et al., 2019; Rossmann et al., 2020). Our results show an overall different microbial layout in the three compartments analyzed and, for bacterial and fungal communities, the differences involving richness, diversity and relative composition. All comparison analyses performed across the 36 bacterial and the 36 fungal samples showed that only the factor compartment explained their separation in three groups corresponding to bulk soil, rhizosphere and root endosphere (*P* < 0.001). In accordance with this premise, the effect of the condition factor on the composition of the microbial communities within each compartment was analyzed. Our results are in agreement with the description of the existence of at least three distinct microbiomes thriving at the root-soil interface (Bulgarelli et al., 2012; Hirsch and Mauchline, 2012). Based on Shannon index estimations, we have observed that the bacterial diversity within each compartment was always higher than that recorded for fungi. We have also observed a decrease of bacterial and fungal diversity from bulk soil to root endosphere of wheat plants, as previously described in microbiome studies from different plants and crop systems (Yamamoto et al., 2018; Ding et al., 2020). The microbial diversity differences detected in wheat plants were accompanied by different composition pictures in relative abundance at the different taxonomical levels investigated (phylum, family, and genus) in the three microhabitats. These observations are in agreement with previous reports that indicate the major role of plants in shaping the composition of each compartment (Vandenkoornhuyse et al., 2015; D’Amico et al., 2018; Rossmann et al., 2020) which should be considered separately.

Our study shows that the bacterial dominant taxa within each compartment were the phyla Proteobacteria and Actinobacteria and that other phyla present across samples differed in relative abundance among compartments. A dominance of Proteobacteria, Acidobacteria and Actinobacteria has been observed in rhizosphere of landraces and modern varieties of wheat (Rossmann et al., 2020). Our data indicate an enrichment in Proteobacteria, Bacterioidetes and Actinobacteria in root endosphere samples. A similar behavior has been found in the root endosphere of grapevines (D’Amico et al., 2018). Results also show a decrease in Acidobacteria, Gemmatimonadetes, Chloroflexi, Verrucomicrobia, Planctomycetes and Cyanobacteria from bulk soil to root endosphere. This should come as no surprise, since the increased number of microbiome studies available (Kavamura et al., 2020; Rossmann et al., 2020) suggests that the particular conditions of every study impact on the microbial communities outcome.

After ALDEx2 and LEfSe analyses, our study discriminated bacterial genera with tropism toward wheat microecosystems. We have seen that *Kaistobacter* was significantly increased in bulk soil, whereas *Flavobacterium*, *Rhizobium* or *Devosia* were overrepresented in the rhizosphere, and *Sphingomonas* in rhizosphere and root endosphere. *Kaistobacter* has been described as one of the most abundant bacterial genera in soil globally (Delgado-Vaquerizo et al., 2018), including those from wheat crops (Schlatter et al., 2019; Zhou et al., 2020). It is not surprising the abundance of *Rhizobium* and *Devosia* close to the root system as they are rhizobacteria with a symbiotic lifestyle with plants (Zhou et al., 2020). In addition, it has been reported the use of antagonistic *Sphingomonas* for the biological control of wheat pathogens (Wachowska et al., 2013). In this sense, diseased wheat was not observed in our field trial. The inclusion of a fallow period of 1 year between barley and wheat crop seasons could have had a positive effect on the maintenance of the plant health status.

Although no significant differences in bacterial abundance were detected among the four conditions when the whole set of samples was compared, there were bacterial taxa showing differential abundance when pairwise comparisons of conditions within a given compartment were performed by ALDEx2, which also includes possibility of doing effect size and significance testing to identify features that are different between groups (Fernandes et al., 2013). Additionally, overrepresented bacterial genera in one of the tested conditions in each compartment were detected in a LEfSe analysis. Considering both approaches, the largest number of differentially abundant taxa was recorded in bulk soil (17 genera) while in the rhizosphere and root endosphere there were three and four, respectively. In general, many differential taxa were recorded as unclassified genus and this is clearly because the generic boundaries in many soil-borne microbes with importance in agriculture (i.e., plant pathogens, biocontrol agents) are still poorly defined. Most of the changes observed in bulk soil were due to the application of CAN top dressing alone or in combination with strain T34. In this sense, the use of CAN top dressing seems to reduce the levels of Actinobacteria but it also increases the levels of *Kaistobacter* together with FCB and PVC bacteria. Although the abundance levels of many taxa were similar for the conditions C2 and C4 (i.e., *Flavisolibacter*, *Kaistobacter*) (Figure 4B), some of them showed no significant differences for the C1–C4 pairwise comparison (i.e., *Rubrobacter*, g_unclassified.f_Sphingomonadaceae), as a result of the enormous variability shown by samples of the C4 condition. ALDEx2 results showed that the application of strain T34 (C3) did not lead to significant changes of bacterial genera in bulk soil compared to the control (C1). This is a confirmation of harmlessness of the use of *Trichoderma* against soil bacteria. Nevertheless, the application of strain T34 was associated by a LEfSe analysis to increased levels of 11 taxa including members of Rhizobiales and Actinobacteria, but also with a reduction of 12 taxa among which are other members of Actinobacteria and *Bacillus* (Figure 5B). In any case, such reductions are not exclusive to apply strain T34 since they can also be associated to the implementation of CAN. Similar to what was observed in the bulk soil, most of the changes detected by ALDEx2 in the rhizosphere are associated with the CAN top dressing and the absence of significance of some taxa in C4, with levels similar to those of C2, may be due to the high abundance variability of the C4 samples (Figure 4C, i.e., *Agrobacterium*). The progressive increase of rhizobia toward the wheat rhizosphere seems to be helped by the application of CAN top dressing. Contrary to what happens in bulk soil, strain T34 applied alone or combined with CAN top dressing seemed to affect the abundance levels of some taxa, although some of the increases, such as those of *Dyadobacter* and a member of Actinomycetales, would be consequence of the combination of both treatments.

It has been reported that inorganic N fertilization can negatively affect wheat rhizosphere bacterial communities (Kavamura et al., 2018), but we have detected that CAN top dressing application was associated with increased levels of *Kaistobacter*, *Gemmatimonas, Flavisolibacter* or *Aeromicrobium* in bulk soil, and *Agrobacterium*, *Devosia* and *Luteolibacter* in rhizosphere. However, similarly to Kavamura et al. (2018), we observed for the condition C2 a reduction in the levels of several genera of bacteria (*Brevibacillus*, *Cellulomonas*, *Rubrobacter*, *Streptomyces*, or *Haliangium*) in bulk soil. Many of these top dressing-impacted genera contain plant beneficial microorganisms used as biocontrol agents and biofertilizers (Bargaz et al., 2018; Begum et al., 2019). Interestingly, the increased levels of biocontrol agents in bulk soil, such as *Rubrobacter*, *Streptosporangium*, and *Haliangium*, were negatively affected by CAN top dressing, and they could be associated with the application of strain T34. As a positive feature of the use of strain T34, it should be noted that the C2-negatively affected strict anaerobic mollicutes *Asteroleplasma* was also favored in its colonization of rhizosphere and endosphere by the application of *Trichoderma*.

Our fungal approach indicates that Ascomycota and Basidiomycota were the most frequent phyla in the wheat microbiome. Although relative abundance data also indicate that Olpidiomycota was the phylum significantly increased in rhizosphere, Ascomycota was overrepresented in root endosphere, and Mortierellomycota and Chytridiomycota were overrepresented in bulk soil. Interestingly, the phylum Chytridiomycota was not present in root endosphere samples and the phylum Glomeromycota was only detected in one of the endosphere samples as it would be expected for AMF and zoospore-forming fungi. A recent study has reported that fungal communities of the wheat rhizosphere are dominated by Ascomycota, followed by Chytridiomycota and Basidiomycota (Rossmann et al., 2020), and it has been reported that saprophytic fungal genera are frequent in the rhizosphere of different crops, including wheat, and that root pathogens are abundant in the wheat rhizosphere (Schlatter et al., 2019). We have observed from ALDEx2 analysis performed at the genus level that saprophytic fungi such as *Mortierella, Solicoccozyma* and *Trichoderma* are overrepresented in bulk soil, and the pathogenic *Zymoseptoria* in the rhizosphere. Likewise, *Fusarium* was also increased in bulk soil and even though some species of *Fusarium* are pathogenic to wheat, the absence of disease in our field assay would be supporting that an important amount of the detected fusaria could be not detrimental or even beneficial for the crop. In addition, the absence of disease could be a consequence of the observed presence of bacteria and fungi with potential activity of biocontrol, as occurs with *Kaistobacter*, *Streptomyces*, *Pseudomonas, Sphingomonas*, and *Trichoderma* (Jung et al., 2013; Liu et al., 2016; Mehrabi et al., 2016; Rubio et al., 2019). Moreover, as indicated above, such absence could be also related to the cultivation history of the experimental field, since the wheat crop was preceded by fallow land. Our data show a comprehensive picture of the significant impact of factor compartment in the relative abundance of fungal taxa observed in wheat, it being also in agreement with the idea of the plant modulating microbial communities assemblage (Bulgarelli et al., 2012; Hirsch and Mauchline, 2012). As it could be expected for a field trial performed under high basal N fertilization, *Mortierella* and *Solicoccozyma*, were amongst the genera with increased abundance in bulk soil detected by LEfSe, and they were previously associated to soil subjected to chemical N fertilization (Ding et al., 2020).

As in the bacterial study, there were fungal taxa showing differential abundance within a given compartment after ALDEx2 analysis and the largest number of taxa with differential abundance was found in bulk soil. Contrary to that observed in the bacterial analysis, several fungal taxa were identified as differentially abundant in the condition C3, this indicating that fungi are affected in a greater extent than bacteria by the application of strain T34. Our results are indicative of the enormous variability of soil fungal systems and their dependence on the treatment applied. In this sense, the changes in abundance observed for several taxa in bulk soil and rhizosphere seem to be a consequence of the applications of CAN top dressing (i.e., *Conocybe*, *Zymoseptoria*), strain T34 (*Trichoderma*) or their combination (*Alternaria*, unclassified.o_Mortierellales). Particularly, *Conocybe* and *Alternaria* have been proposed as bioindicators of intensive crop soils subjected to N fertilization (Schöps et al., 2018). Our results show that the strain T34 increased the levels of the *Trichoderma* genus in bulk soil while the single or T34-combined application of CAN top dressing reduced those levels in the rhizosphere. In this sense, several reports have shown that microbial communities of different crops are affected by the introduction of a *Trichoderma* strain (Umadevi et al., 2017; Schöps et al., 2018; Singh et al., 2018) but the opposite has been also described (Ganuza et al., 2019; Wang et al., 2019), illustrating that the increased availability of nutrients is not the sole mechanism which explains this fact. In other way, it is well known that the use of AMF inoculants increases the production of many crops, including wheat under drought stress conditions (Begum et al., 2019), and we have previously reported that strain T34 facilitates the access of AMF to non-host Brassicaceae *Arabidopsis* and rapeseed roots, with increased production (Poveda et al., 2019). Moreover, the AMF *Claroideoglomus* has been described as an abundant genus in the wheat rhizosphere under intensive chemical N fertilization (Sommermann et al., 2018). Our study shows that *Claroideoglomus* levels in bulk soil were negatively affected by the application of CAN top dressing, strain T34 or both. However, the single application of T34 favored the presence of *Claroideoglomus* in the rhizosphere. Thus, the trophic dependencies derived from N fertilization and *Trichoderma* application (Rubio et al., 2019) impact on the AMF abundance in wheat bulk soil and rhizosphere microbiome. Two beneficial fungi such as the yeast *Sporobolomyces* and *Mortierella* were increased in the endosphere by CAN top dressing application. This result was to be expected since *Mortierella* is considered a root-associated fungal metacommunity hub (Wani et al., 2017) proposed as soil fungal bioindicator after chemical N fertilization (Ding et al., 2020) and it has been reported that CAN supports the growth of *Sporobolomyces* when colonizing wheat plants (Frossard et al., 1983).

## Conclusion

Although the factor top dressing increased wheat quality parameters, neither CAN applications nor seed-inoculated strain T34 impacted the crop yield in our field trial. The significant differences observed in bacterial and fungal richness, diversity and relative composition among bulk soil, rhizosphere and root endosphere show a specific trophic behavior in these three wheat microhabitats. Bulk soil overrepresented bacterial and fungal genera here recorded are microbes associated to soils with an abuse of chemical N fertilization history, and most changes in microbial abundance associated to conditions occurred in this compartment. The single or strain T34-combined application of CAN top dressing affected to a greater extent the bulk soil bacterial levels than the use of T34 alone. When combined, CAN top dressing played a more decisive role in the bacterial microbiome of wheat than strain T34. Particularly, the three tested treatments reduced the levels of the AMF *Claroideoglomus* in bulk soil, although strain T34 increased the rhizhosphere abundance of this mycorrhizal fungus as well as that of plant beneficial rhizobacteria. The fungal microbiome of wheat bulk soil and rhizosphere was notably affected and to varying degrees by any of the three treatments assayed. Interestingly, bacterial and fungal genera negatively affected by CAN top dressing were increased in their bulk soil and rhizosphere levels after the implementation of strain T34. The results obtained can provide the basis for future trials using lower doses of inorganic N fertilization aimed at favoring a specific microbiome able to allow acceptable agronomic traits with less environmental impact. Further studies focused on isolating potential biofertilizers from the wheat rhizosphere to be used as synthetic communities to favor microbiota recruitment in exhausted crop soils are considered important. The application of microbial synthetic communities particularly selected for wheat cropping could help to modulate root microbiomes in order to sustain plant health and consequently productivity.

## Data Availability Statement

The raw sequencing data (16S and ITS rRNA gene fastq files) are publicly available in the NCBI Sequence Read Archive (SRA), Bioproject PRJNA639567 (bacterial libraries, run numbers SRR12018013 to SSR12018048; and fungal libraries, SSR12023978 to SSR12024013).

## Author Contributions

RH and EM conceived the research. MI, MR, VH-R, CN, and RH performed the experiments. MI, MR, MM-D, EM, and RH analyzed the data. MI, MM-D, and AM prepared the tables and figures. EM and RH contributed to reagents, materials, and analysis tools. EM and RH wrote the manuscript with all authors contributing to the discussion of the data.

## Conflict of Interest

The authors declare that the research was conducted in the absence of any commercial or financial relationships that could be construed as a potential conflict of interest.

## Supplementary Material

Supplementary Figure 1 Schematic diagram of the wheat field trial. **(A)** Layout of the field trial showing the randomized distribution of the four conditions (C1–C4) and their three biological replicates. **(B)** Details of a plot with its 6 beds (white boxes) and the five spots where the soil (S1–S5) and plant samples (P1–P5) were collected.

Supplementary Figure 2 Bacterial taxa overrepresented in wheat crop plant compartments (bulk soil, rhizosphere, endosphere) after LEfSe analysis at the genus level. Taxonomic cladogram showing differences from phylum to genus level **(A)**, and bacterial taxa with LDA > 4 (*P* < 0.05) **(B)** (*n* = 3). In the cladogram, dot size is proportional to taxon abundance and letters refer to the taxa listed on the right. In the barplot, black boxes refer to the taxa identified by both LEfSe and ALDEx2 analyses.

Supplementary Figure 3 Fungal taxa overrepresented in wheat crop plant compartments (bulk soil, rhizosphere, and root endosphere) after LEfSe analysis at the genus level. Taxonomic cladogram showing differences from phylum to genus level **(A)**, and fungal taxa with LDA > 4 (*P* < 0.05) **(B)** (*n* = 3). In the cladogram, dot size is proportional to taxon abundance and letters refer to the taxa listed on the right. In the barplot, black boxes refer to the taxa identified by both LEfSe and ALDEx2 analyses.

Supplementary Table 1 Information about statistics and quality of 16S sequences.

Supplementary Table 2 Information about statistics and quality of ITS sequences.

Supplementary Table 3 Bacterial OTU lists, assigned at phylum and family levels, showing differences in percentage of relative abundance among bulk soil, rhizosphere and root endosphere samples of wheat crop plants (ALDEx2 analysis, *P* < 0.05, *n* = 3).

Supplementary Table 4 Bacterial abundance statistical analysis inferred from an ALDEx2 compositional analysis at the genus level. Relative abundance is calculated as mean proportion. Pairwise comparisons among compartments (bulk soil, rhizosphere, endosphere) and among conditions (C1, C2, C3, C4) are presented (*n* = 3).

Supplementary Table 5 Bacterial taxa enriched in compartments (bulk soil, rhizosphere, endosphere) and conditions (C1, C2, C3, C4) within a given compartment, inferred from a LEfSe analysis at the genus level. LDA threshold score > 4.0 for compartments and >2.0 for conditions (*P* < 0.05) (*n* = 3).

Supplementary Table 6 Fungal OTU lists, assigned at phylum and family levels, showing differences in percentage of relative abundance among bulk soil, rhizosphere and root endosphere samples of wheat crop plants (ALDEx2 analysis, *P* < 0.05, *n* = 3).

Supplementary Table 7 Fungal abundance statistical analysis inferred from an ALDEx2 compositional analysis at the genus level. Relative abundance is calculated as mean proportion. Pairwise comparisons among compartments (bulk soil, rhizosphere, endosphere) and among conditions (C1, C2, C3, C4) are presented (*n* = 3).

Supplementary Table 8 Fungal taxa enriched in compartments (bulk soil, rhizosphere, endosphere) and conditions (C1, C2, C3, C4) within a given compartment, inferred from a LEfSe analysis at the genus level. LDA threshold score > 4.0 for compartments and >2.0 for conditions (*P* < 0.05) (*n* = 3).
