# Supplementary figures and images for: Effect of Inorganic N Top Dressing and Trichoderma harzianum Seed-Inoculation on Crop Yield and the Shaping of Root Microbial Communities of Wheat Plants Cultivated Under High Basal N Fertilization
Source: Front Plant Sci. 2020 Oct 23;11:575861. doi: 10.3389/fpls.2020.575861 (PMC7644891; doi:10.3389/fpls.2020.575861)

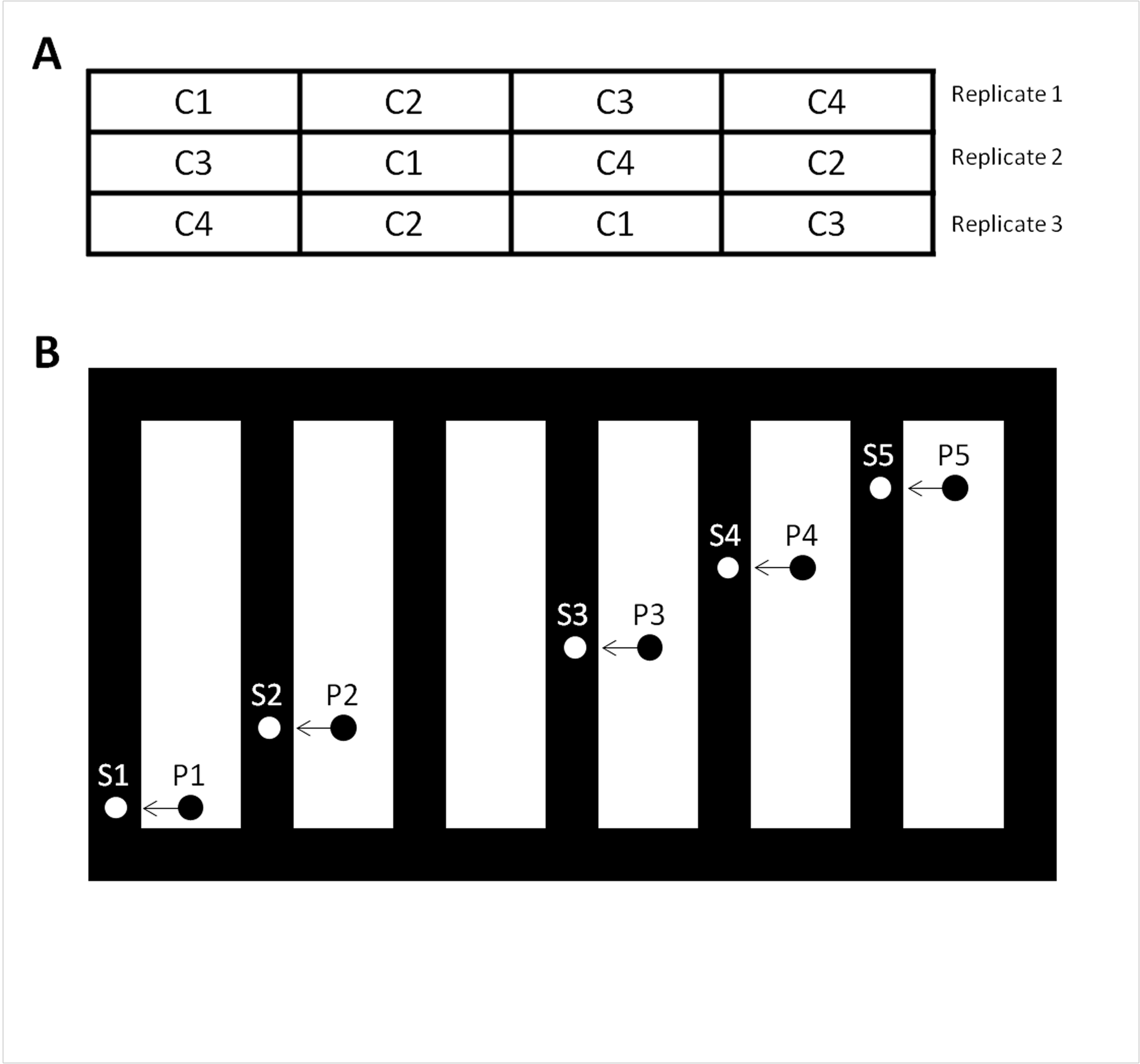

Supplement: Supplementary Figure 1 — Schematic diagram of the wheat field trial. (A) Layout of the field trial showing the randomized distribution of the four conditions (C1–C4) and their three biological replicates. (B) Details of a plot with its 6 beds (white boxes) and the five spots where the soil (S1–S5) and plant samples (P1–P5) were collected. [file Image_1.TIF]

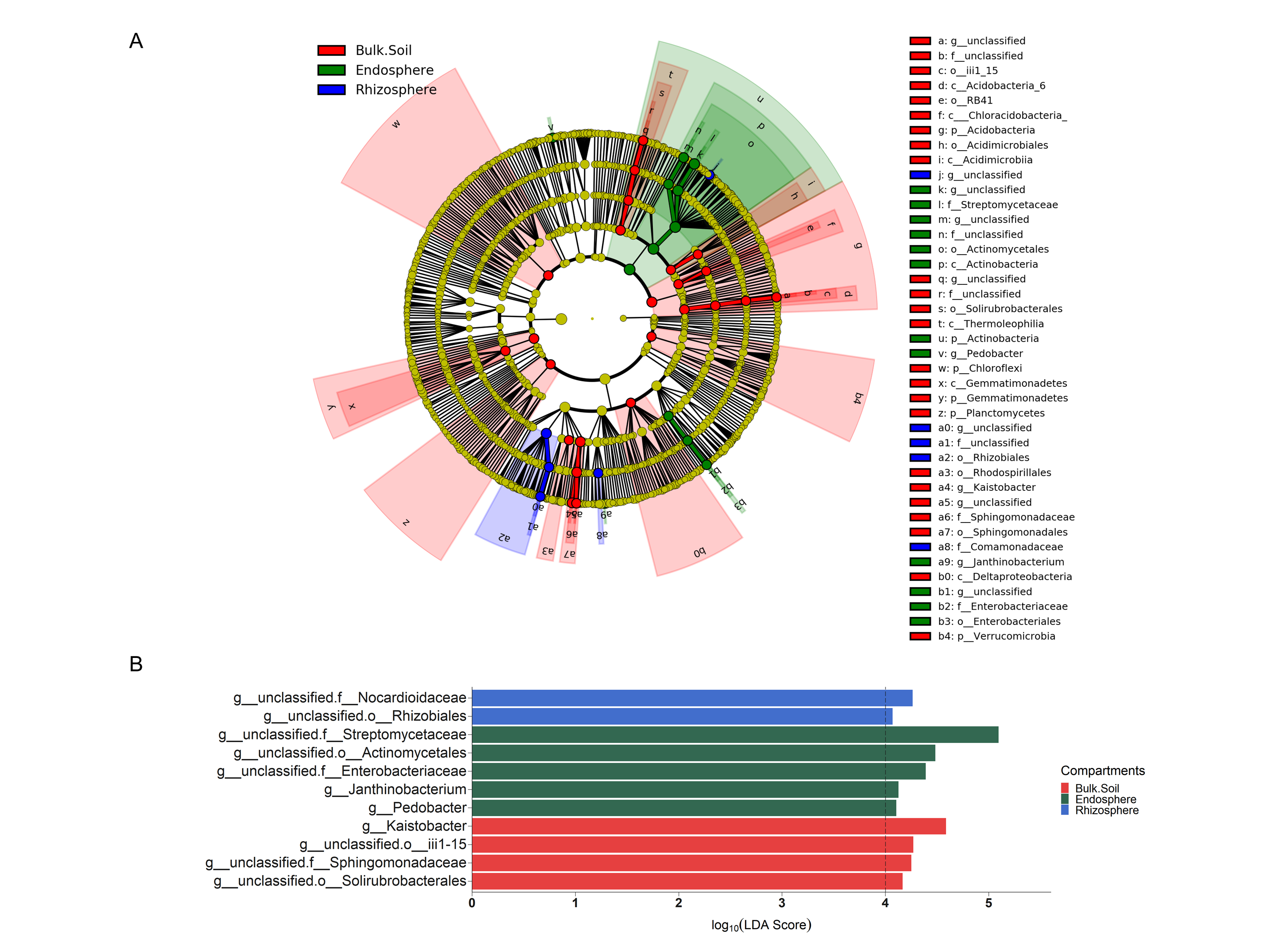

Supplement: Supplementary Figure 2 — Bacterial taxa overrepresented in wheat crop plant compartments (bulk soil, rhizosphere, endosphere) after LEfSe analysis at the genus level. Taxonomic cladogram showing differences from phylum to genus level (A), and bacterial taxa with LDA > 4 (P < 0.05) (B) (n = 3). In the cladogram, dot size is proportional to taxon abundance and letters refer to the taxa listed on the right. In the barplot, black boxes refer to the taxa identified by both LEfSe and ALDEx2 analyses. [file Image_2.TIF]

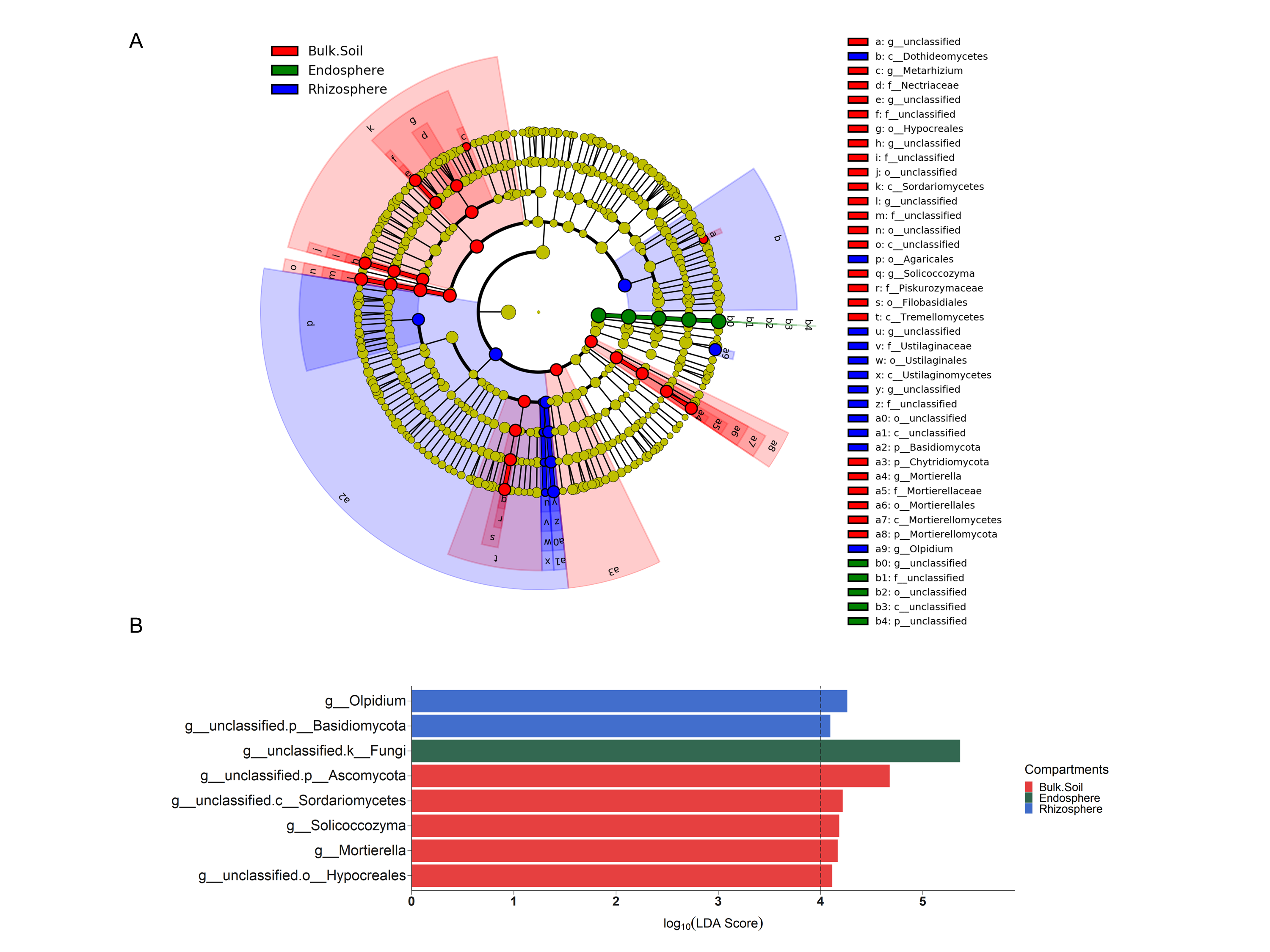

Supplement: Supplementary Figure 3 — Fungal taxa overrepresented in wheat crop plant compartments (bulk soil, rhizosphere, and root endosphere) after LEfSe analysis at the genus level. Taxonomic cladogram showing differences from phylum to genus level (A), and fungal taxa with LDA > 4 (P < 0.05) (B) (n = 3). In the cladogram, dot size is proportional to taxon abundance and letters refer to the taxa listed on the right. In the barplot, black boxes refer to the taxa identified by both LEfSe and ALDEx2 analyses. [file Image_3.TIF]
